# Pressurized IntraPeritoneal Aerosol Chemotherapy (PIPAC) Applied to Platinum-Resistant Recurrence of Ovarian Tumor: A Single-Institution Experience (ID: PARROT Trial)

**DOI:** 10.1245/s10434-023-14648-0

**Published:** 2023-12-15

**Authors:** Giuseppe Vizzielli, Maria Teresa Giudice, Federica Nardelli, Barbara Costantini, Vanda Salutari, Frediano Socrate Inzani, Gian Franco Zannoni, Vito Chiantera, Andrea Di Giorgio, Fabio Pacelli, Anna Fagotti, Giovanni Scambia

**Affiliations:** 1 Department of Medicine, University of Udine, Udine, Italy; 2 Clinic of Obstetrics and Gynecology, “Santa Maria della Misericordia” University Hospital, Azienda Sanitaria Universitaria Friuli Centrale, Udine, Italy; 3 Gynecologic Oncology Unit, Department of Woman, Child and Public Health, Fondazione Policlinico Universitario Agostino Gemelli IRCCS, Rome, Italy; 4 Saint Camillus International, University of Health Sciences, Rome, Italy; 5 Anatomic Pathology Unit, Department of Molecular Medicine, University of Pavia, Pavia, Italy; 6 Gynecopathology and Breast Pathology Unit, Department of Woman, Child and Public Health, Fondazione Policlinico Universitario Agostino Gemelli IRCCS, Rome, Italy; 7 Catholic University of Sacred Heart, Rome, Italy; 8 Department of Gynecologic Oncology, ARNAS “Civico – Di Cristina – Benfratelli”, Department of Health Promotion, Mother and Child Care, Internal Medicine and Medical Specialties (PROMISE), University of Palermo, Palermo, Italy; 9 Surgical Unit of Peritoneum and Retroperitoneum, Fondazione Policlinico Universitario Agostino Gemelli IRCCS, Rome, Italy

**Keywords:** Ovarian cancer, Peritoneal carcinomatosis, Laparoscopy, Platinum-resistant recurrence, PIPAC, Surgery

## Abstract

**Background:**

We aimed to investigate the therapeutic efficacy and safety of Pressurized IntraPeritoneal Aerosol Chemotherapy (PIPAC) in platinum-resistant recurrence of ovarian cancer and peritoneal carcinomatosis, while our secondary endpoint was to establish any changes in quality of life estimated via the EORTC QLQ-30 and QLQ-OV28 questionnaires.

**Methods:**

In this monocentric, single-arm, phase II trial, women were prospectively recruited and every 28–42 days underwent courses of PIPAC with doxorubicin 2.1 mg/m^2^ followed by cisplatin 10.5 mg/m^2^ via sequential laparoscopy.

**Results:**

Overall, 98 PIPAC procedures were performed on 43 women from January 2016 to January 2020; three procedures were aborted due to extensive intra-abdominal adhesions. The clinical benefit rate (CBR) was reached in 82% of women. Three cycles of PIPAC were completed in 18 women (45%), and 13 (32.5%) and 9 (22.5%) patients were subjected to one and two cycles, respectively. During two PIPAC procedures, patients experienced an intraoperative intestinal perforation. There were no treatment-related deaths. Nineteen patients showed no response according to the Peritoneal Regression Grading Score (PRGS) and 8 patients showed minor response according to the PRGS. Median time from ovarian cancer relapse to disease progression was 12 months (95% confidence interval [CI] 6.483–17.517), while the median overall survival was 27 months (95% CI 20.337–33.663). The EORTC QLQ-28 and EORTC QLQ-30 scores did not worsen during therapy.

**Conclusions:**

PIPAC seems a feasible approach for the treatment of this subset of patients, without any impact on their quality of life. Since this study had a small sample size and a single-center design, future research is mandatory, such as its application in addition to systemic chemotherapy.

**Supplementary Information:**

The online version contains supplementary material available at 10.1245/s10434-023-14648-0.

Peritoneal carcinomatosis is a common condition in epithelial ovarian cancer and affects about 75% of women at the time of first diagnosis. Despite extensive frontline surgery and standard adjuvant chemotherapy with platinum-based treatments, around 80% of women diagnosed at an advanced stage will experience disease recurrence.^1^

Women showing recurrent ovarian cancer more than 6 months after first-line chemotherapy are considered to have *platinum-sensitive* disease. In this context, as shown in the final analysis of the randomized controlled study DESKTOP III, highly selected patients with isolated relapse of disease can benefit from cytoreductive surgery with respect of chemotherapy alone.^2–7^ Furthermore, the recent introduction of the minimally invasive approaches may further reduce the surgical morbidities in this subset of patients.^8,9^

On the other hand, recurrent ovarian cancer patients showing relapse within the first 6 months after completion of platinum-based chemotherapy are defined as *platinum-resistant*. According to the most recent guidelines, women experiencing platinum-resistant relapse, regardless of the extension and pattern of disease presentation, are offered salvage systemic chemotherapy as the only recommended therapeutic option, given the short survival.^10^ Even if some retrospective studies have demonstrated that secondary cytoreductive surgery (SCS) prolongs post-relapse survival compared with chemotherapy alone in patients with isolated platinum-resistant recurrent ovarian cancer,^11,12^ palliative intravenous chemotherapy remains the only option in this specific subgroup of women.

However, the efficacy of intravenous chemotherapy against peritoneal carcinomatosis could be affected by several factors, including poor blood supply within solid nodules^13^ and elevated intratumoral fluid pressure.^14^ In this context, some initial promising experiences have been published regarding the role of surgery combined with hyperthermic intraperitoneal chemotherapy (HIPEC) in treating peritoneal relapse within 6 months from completion of primary platinum-based chemotherapy by means of improving the locoregional control of disease.^15–18^ However, many of these studies are focused on resectable disease, leaving systemic chemotherapy as the only option in cases of peritoneal carcinomatosis.

Over the last decade, a new technique has been offered to patients affected by platinum-resistant recurrence of ovarian cancer and peritoneal disease, which allowed increased bioavailability of drugs compared with conventional HIPEC. Pressurized intraperitoneal aerosol chemotherapy (PIPAC) combines the benefits of a minimally invasive approach (easier repeat application, lower morbidity, shorter hospital stay) with the pharmacokinetic advantages of intraperitoneal administration (higher intratumoral concentrations, less systemic toxicity) under pressurized vaporization (increased distribution and deeper penetration).^19,20^ This new drug-delivery technique has been increasingly used in cases of peritoneal metastasis, with some authors reporting promising results both in terms of quality of life (QoL)^21^ and local disease control.^22,23^

PIPAC represents an experimental treatment, which is only allowed in controlled clinical trials and is meant to demonstrate an increase in drug effectiveness related to the physical properties of pressure and gas. Scientific evidence supporting PIPAC outside an experimental setting is still lacking.^24^

In this study, our co-primary endpoint was to investigate the feasibility of PIPAC, in terms of therapeutic efficacy and safety, using cisplatin and doxorubicin in an homogeneous setting of women with platinum-resistant recurrence of ovarian cancer receiving up to two lines of chemotherapy.^25^ Our secondary endpoint was to determine QoL on the basis of the European Organization for Research and Treatment of Cancer (EORTC) QOL questionnaires (QLQ-C30 and QLQ-OV28), before and after application of the PIPAC cycle.^26,27^

## Methods

From January 2016 to January 2020, patients affected by platinum-resistant recurrence of ovarian tumor, with an Eastern Cooperative Oncology Group (ECOG) performance status of ≤ 3, and after one or two previous regimens of chemotherapy, were prospectively recruited at Fondazione Policlinico Universitario “Agostino Gemelli” IRCCS, Rome, Italy. Before admission to the experimental protocol, each patient was discussed in the multidisciplinary tumor board and the indication for PIPAC was decided according to the patients’ clinical conditions and history of disease. A standard preoperative work-up (i.e., chest x-ray, blood tests) was performed before each procedure. Chronic and uncontrolled systemic disorders, bowel obstruction, and/or extraperitoneal metastasis were considered contraindications for PIPAC administration. This study was conducted in accordance with the declaration of Helsinki, was approved by the local Ethical Committee (Prot. N.80, 22/12/2015), and was registered at ClinicalTrials.gov (ClinicalTrials.gov identifier: NCT02735928). Trial management and progress were monitored by an independent data monitoring committee (i.e., Data Collection Facility) and the SPIRIT (Standard Protocol Items: Recommendations for Interventional Trials) statement was followed as close as possible.^28^ All patients signed an informed consent form at least 1 day prior to enrollment.

### Surgical Procedure

The PIPAC procedure was performed according to the previously published consensus guidelines of the ISSPP-PIPAC study group.^29–31^ Briefly, after insufflation of 12 mmHg of capnoperitoneum at 37°C, two balloon trocars (Applied Medical^®^) were placed. Explorative laparoscopy was performed as usual at our institution and the Peritoneal Carcinomatosis Index was determined, according to Fagotti’s score.^32,33^ Parietal biopsies were taken and ascites were removed. A nebulizer (MIP, Reger Medizintechnik, Rottweil^®^) was connected to a high-pressure injector (Injektron 82M, MedTron, Saarbruecken^®^) and was inserted into the abdomen through a trocar. A pressurized aerosol containing cisplatin (Hexal, Barleben) at a dose of 10.5 mg/m^2^ body surface in 150 mL NaCl 0.9% followed by doxorubicin at a dose of 2.1 mg/m^2^ body surface in a 50 mL NaCl 0.9% solution was immediately applied via a nebulizer, according to a previous study.^34^ The system was then kept in a steady state for 30 min (i.e., *application time*). At the end of the procedure, a peritoneal biopsy was performed, with positioning of a metal landmark clip nearby. The landmark clip was used in subsequent procedures to perform the biopsy at the same point and limit as much as possible as the heterogeneity of the pathologist’s reading on the pathological finding. Toxic aerosol was exhausted over a closed system and the trocars were removed. The PIPAC procedure was repeated after 4–6 weeks until progression or limiting toxicity. Adverse events were graded according to the Memorial Sloan Kettering Cancer Center surgical grading system.^35^ In the meantime, the QOL questionnaires were administered to assess the QOL of cancer patients after any cycle of PIPAC.

Despite its known limited accuracy in detecting small peritoneal lesions and the involvement of the small bowel/mesentery, the Response Evaluation Criteria in Solid Tumors (RECIST) version 1.1,^36^ through contrast-enhanced computed tomography (CT), remains the standard imaging modality in the assessment of PC. However, we defined the clinical benefit rate (CBR) as the percentage of advanced cancer patients who achieved complete response (CR), partial response (PR), or at least 6 months of stable disease (SD) as a result of therapy, according to RECIST 1.1 criteria.^37^

Since CT shows only limited diagnostic accuracy and underestimates the real extent of peritoneal carcinomatosis,^38^ we used laparoscopic assessment to complement radiological data and to assess the change in tumor burden. The gold standard for measuring the extent of peritoneal carcinomatosis is the Peritoneal Cancer Index (PCI),^39^ but in our institution we routinely use Fagotti’s score to evaluate the distribution and volume of the abdominal disease in patients with a first diagnosis of ovarian cancer. Thus, we decided to also apply Fagotti’s scoring method in this population of patients affected by platinum-resistant ovarian cancer.

*A priori,* as an ancillary report and in addition to the standard chemotherapy treatment response criteria (i.e., RECIST 1.1 criteria), we applied the following laparoscopic definitions for macroscopic response during laparoscopy.

*Complete response (CR):* Fagotti’s score = 0, with negative histology of at least three peritoneal biopsies of suspect nodules.

*Partial response (PR):* At least two parameters had a decrease in Fagotti’s score.

*Progressive disease (PD):* At least one parameter had an increase in Fagotti’s score. The appearance of one or more new lesions is also considered progression.

*Stable disease (SD):* Neither sufficient shrinkage to qualify for PR nor sufficient increase to qualify for PD, taking as reference Fagotti’s score.

However, the laparoscopic evaluation was only used as an ancillary report and no clinical decision was based on it.

### Pathological Evaluation

The Peritoneal Regression Grading Score (PRGS) by Solass et al.^40^ was used to grade the regressive changes in the obtained biopsies and/or surgical specimens. The evaluation of PRGS is defined as follows:

- *Grade 1:* Complete response (absence of tumor cells).
- *Grade 2:* Major response (major regression features, few residual tumor cells).
- *Grade 3*: Minor response (some regressive features but predominance of residual tumor cells).
- *Grade 4:* No response (tumor cells without any regressive features).

Herein, an increase in PRGS between the first PIPAC (PIPAC1) and the last PIPAC performed in each patient was determined as iPRGS, as similarly described in a previous paper:^41^ iPRGS+ in the case of increased PRGS, and iPRGS− in the case of absence of increased PRGS. Mean PRGS was considered when more than one biopsy was performed during the PIPAC procedure.

### Statistical Analysis

The co-primary endpoints were to establish the feasibility both in terms of efficacy (i.e., CBR) and safety, while the secondary endpoint was to evaluate QoL during its application. All *p*-values were two-tailed and a *p*-value <0.05 was considered statistically significant. The sample size was calculated based on a Simon two-stage design for a phase II study. Based on clinical trials of women with recurrent ovarian cancer undergoing experimental chemotherapy regimens of fewer than two lines of standard chemotherapy and reporting a CBR of 25–60%,^42–44^ we regarded *a priori* a proportion of patients with a CBR of ≥ 40% as proof of efficacy of PIPAC in this patient population, and of < 20% as insufficient to continue the assessment. Assuming a risk of *α* = 0.05 (type I error) and *β* = 0.20 (type II error), we needed to include 36 patients; considering a withdrawal rate of at least 10%, a total of 43 women were enrolled.

Analysis was by intention to treat and was performed using non-parametric tests since data were not normally distributed. Values are given as medians. Survival was modeled in a Kaplan–Meier survival curve. With the term of time to progression (TTP) we mean median time from the ovarian cancer relapse diagnosis to disease progression. Overall survival was defined as the time from the ovarian cancer relapse diagnosis to last follow-up, and QoL was measured using the EORTC QLQ-C30 and QLQ-OV28 questionnaires, validated tools for assessing QoL in cancer patients.^24,25^ QoL was recorded 1 day before each PIPAC procedure. For evaluation of QoL data, analysis of variance (ANOVA) for repeated measures was used to analyze modifications of QoL measures over time. No imputation was undertaken to deal with missing data at any time-point since there were no missing values.

For statistical analysis, we used NCSS 11.0 software and PASS 15.0 (Power Analysis and Sample Size Software; NCSS, LLC. Kaysville, UT, USA [ncss.com/software/pass]).

## Results

**Fig. 1:**
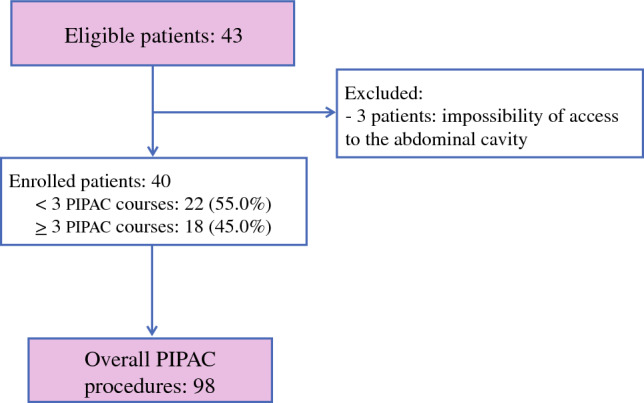
Study flowchart. PIPAC Pressurized IntraPeritoneal Aerosol Chemotherapy

Ninety-eight PIPAC procedures were performed in 43 women from January 2016 to January 2020 (Fig. 1). In only three patients, intraperitoneal therapy was not carried out as a result of impossible access to the abdominal cavity due to the extensive and strong adhesions, with a final feasibility rate of 93%. Patients’ median age was 58.5 years (range 33–70 years). Most patients (88.4%) were affected by ovarian serous carcinoma (36 woman had a high-grade serous carcinoma and two patients had a low-grade serous carcinoma), four women were affected by clear cell cancers and one women was affected by mucinous carcinoma. All patients received fewer than two platinum-based chemotherapy regimens after primary or interval debulking surgery, with a median platinum-free interval of 4 months (range 0–6) (Table 1). At the time of laparoscopy for the PIPAC procedure, the median Fagotti’s score was 10 (range 8–12) and ascites was documented in 13 patients (30.2%). Almost half of the patients underwent three or more cycles of PIPAC therapy (18 patients, 45.0%), while 13 (32.5%) and 9 (22.5%) patients were subjected to only one and two cycles of PIPAC, respectively, due to disease progression documented with a radiological instrumental examination performed for worsening of clinical conditions.

**Table 1:** Demographics and clinical characteristics

Characteristics	Value (range, %)
No. of patients	43
No. of PIPAC procedures	98
Age, years [median (range)]	58.5 (33–70)
BMI, kg/m^2^ [median (range)]	22.7 (17.6–37)
ECOG performance status [median (range)]	1 (0–1)
Previous abdominal surgeries, *n* [median (range)]	2 (1–6)
Tumor histology, *n*	
Serous high grade	36/43
Serous low grade	2/43
Clear cell	4/43
Mucinous	1/43
PFI, months [median (range)]	4 (0–6)
Previous chemotherapy regimens, *n* [median (range)]	1 (1–2)
CA125, IU/mL [median (range)]	92.3 (16–2120)
Fagotti’s score [median (range)]	10 (8–12)
Presence of ascites	13/43
Ascites volume, mL [median (range)]	1900 (200–3500)
Feasibility rate [*n* (%)]	40/43 (93.0)
PIPAC applications, *n*	
One cycle	13
Two cycles	9
Three cycles	16
Four or more cycles	2

*PIPAC* Pressurized IntraPeritoneal Aerosol Chemotherapy, *BMI* body mass index, *ECOG* Eastern Cooperative Oncology Group, *PFI* platinum-free interval

The median operative time was 145 min (range 90–295 min), median estimated blood loss was 5 mL (range 0–10 mL), and the median length of hospital stay was 2 days (range 1–10 days). Early postoperative complications were documented during 8 of the 98 total procedures (8.2%). G2 complications occurred in six patients, including two patients with bowel obstruction that was managed with intravenous hydration and parenteral nutrition, one case of anemia treated with a blood transfusion, two patients with systemic infections, and one patient with abdominal collection that required antibiotic therapy. Two (2%) patients had G3 complications, both with intestinal perforation during open laparoscopic entry, requiring operative laparotomy to repair a lesion and antibiotic therapy during postoperative hospital stay. Thirty-day mortality was not documented. During PIPAC therapy, sclerosis of peritoneal nodules was observed, as well as scarring of the visceral and parietal peritoneum (Table 2).

**Table 2:** Intraoperative, postoperative, and oncological outcomes

Characteristics	Value (range, %)
No. of patients	438
No. of PIPAC procedures	98
Operative time, min [median (range)]	145 (90–295)
Estimated blood loss, mL [median (range)]	5 (0–10)
Intraoperative complications	2 (2.0%)
Small bowel injury	2
Early postoperative complications^a^	
Grade 2	8/98 (8.2%)
Abdominal collection requiring intravenous antibiotic therapy	1
Sepsis requiring intravenous antibiotic therapy	2
Bowel obstruction managed with 3-day intravenous hydration	2
Anemia requiring blood transfusion	1
Grade 3	
Intestinal perforation requiring operative laparotomy	2
Hospital stay [median (range)]	2 (1–10)
Overall benefit (%)	33/40 (82.5)
CTCAE^b^	
> 2 (%)	2/40 (5.0)
Mortality (30 days)	0
Laparoscopical response, evaluable cases [*n* (%)]	27 (67.5)
Stable disease	24 (88.9)
Disease progression	3 (11.1)
Pathological response,^c^ evaluable cases [*n* (%)]	27 (67.5)
Minor response (Grade 3)	8 (29.6)
No response (Grade 4)	19 (70.4)
Follow-up, months	24
Time to progression, months	
From the first PIPAC application [median (range)]	12 (0–33)
From the last PIPAC application [median (range)]	1 (0–11)
Post-relapse survival, months [median (range)]	27 (1–34)
Death of disease [*n* (%)]	28/40 (70.0%)

*PIPAC* Pressurized IntraPeritoneal Aerosol Chemotherapy, *CTCAE* Common Terminology Criteria for Adverse Events

^a^According to the Memorial Sloan Kettering Cancer Center surgical grading system^30^

^b^https://ctep.cancer.gov/protocoldevelopment/electronic_applications/docs/CTCAE_v5_Quick_Reference_8.5x11.pdf (Accessed 9 Mar 2018)^45^

^c^According to the Peritoneal Regression Grading Score (PRGS)^39^

**Fig. 2:**
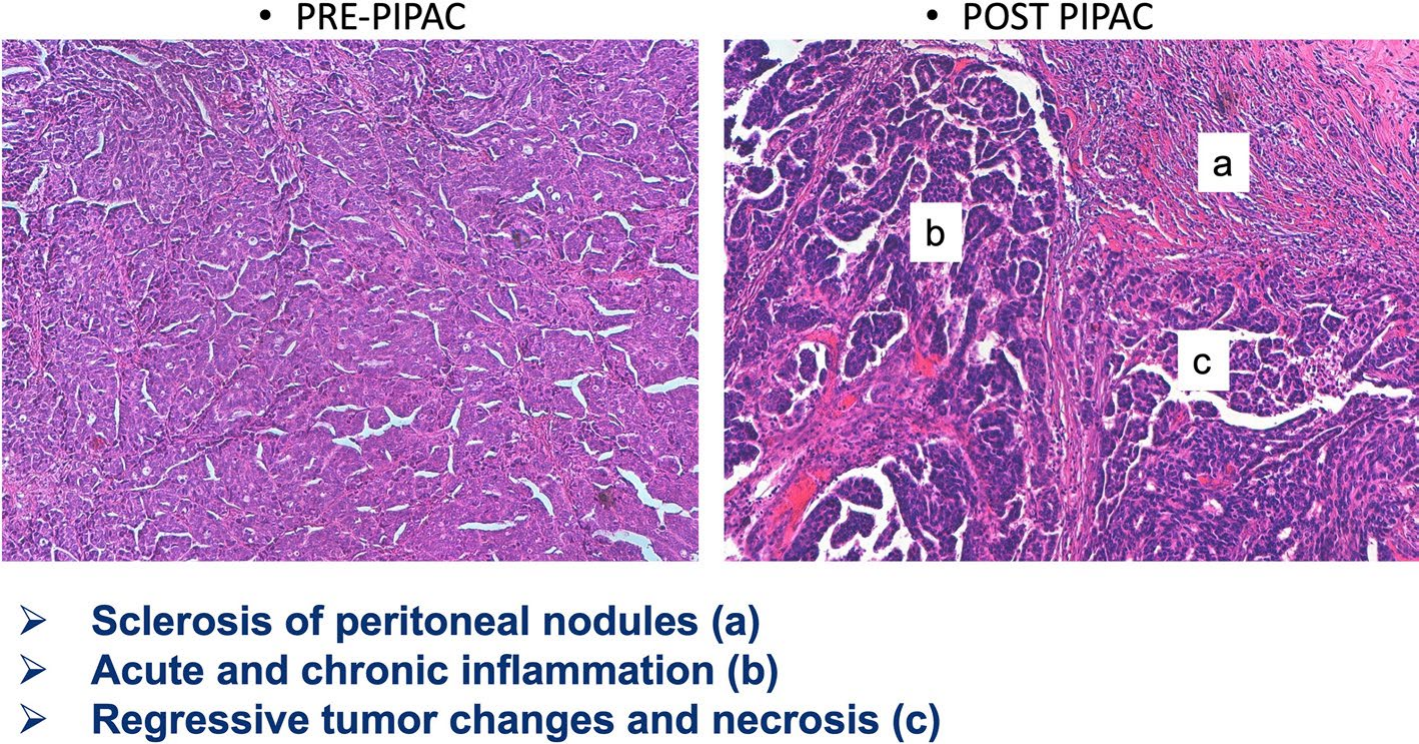
Pathological features before and after PIPAC treatment. PIPAC Pressurized IntraPeritoneal Aerosol Chemotherapy

Corresponding histological specimens taken during the first, second, and third PIPAC application demonstrated regressive tumor changes, fibrosis, and acute and chronic inflammation (Fig. 2). During the PIPAC treatment period, some variations in the PRGS were observed comparing the value assessed on biopsies performed during the first PIPAC procedure and the last biopsy for each patient. In detail, 19 patients showed an increase in PRGS and 8 patients did not show an increase; all 8 women show minor response (grade 3) and no patients showed a decrease in the PRGS. Otherwise, no macroscopic variations of carcinosis were observed, as described by Fagotti’s scores, with respect to histological findings (data not shown).

EORTC QLQ-30 symptom scores did not show any statistical differences, even if nausea/vomiting, appetite loss, dyspnea, emotional functioning, and constipation appeared to slightly decrease during therapy. In addition, physical, cognitive, fatigue, and pain scores increased during therapy. On the QLQ-OV28 symptom score, we also did not observe any statistical difference. However, some abdominal symptoms seemed to slightly increase and, on the other hand, other chemotherapy adverse effects, such as body image and hair loss, appeared to improve during therapy (Electronic Supplementary Tables 1 and 2).

**Fig. 3:**
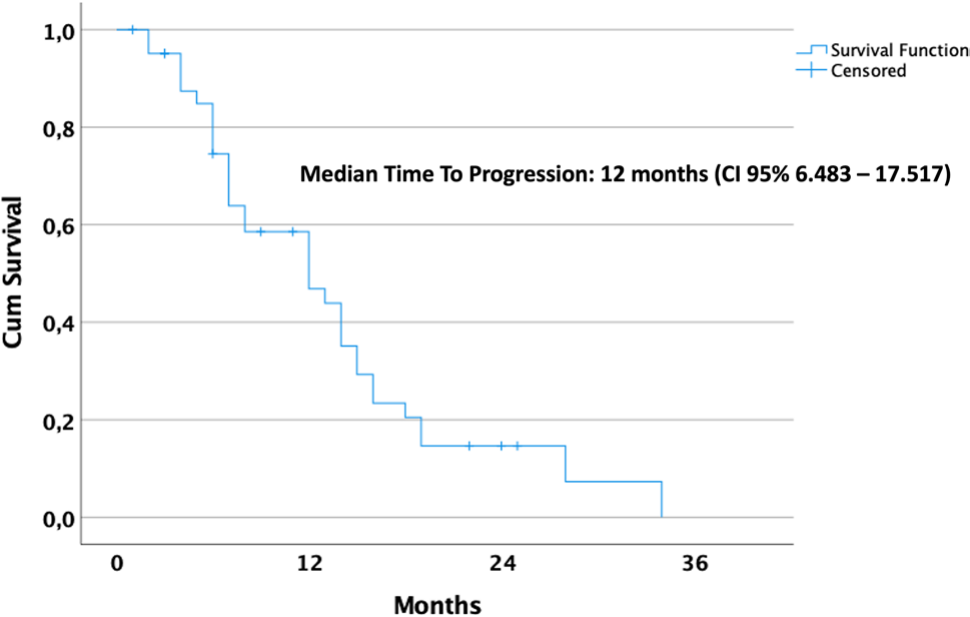
Kaplan–Meier plots for Time To Progression (TTP). Cum Cumulative, CI confidence interval

**Fig. 4:**
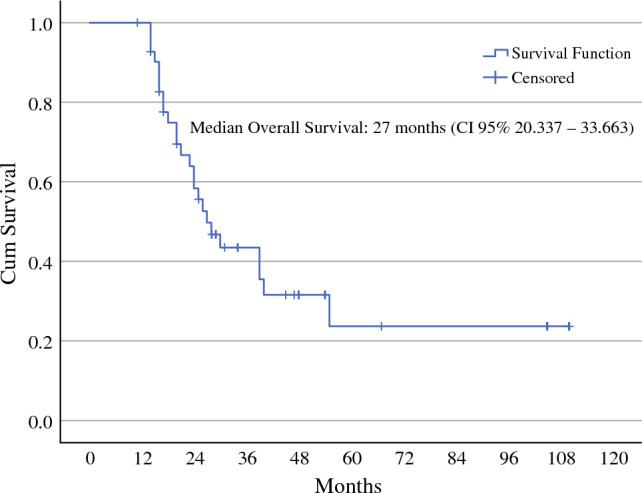
Kaplan–Meier plots for overall survival. Cum cumulative, CI confidence interval

Post-relapse survival and overall survival are shown in Figs. 3 and 4. The median duration of follow-up was 30 months. With a CBR of 82%, the median time from ovarian cancer relapse diagnosis to disease progression (time to progression [TTP]) was 12 months (95% confidence interval [CI] 6.483–17.517), documented by radiological examinations, while the median time from the last PIPAC procedure to disease progression was 1 month (0–6).

Median overall survival was 27 months (95% CI 20.337–33.663). Twenty-eight patients (70%) died as a result of multi-organ failure seen with disease progression at the time of last follow-up.

## Discussion

Large population-based studies have shown that patients with platinum-resistant recurrence of ovarian cancer have poor prognosis, with a median overall survival of 10–12 months.^46^ New treatment modalities are desperately needed and PIPAC-directed therapy has emerged as an option in this subset of women. To our knowledge, this is the first study that tests the feasibility of PIPAC, in terms of efficacy and safety, in an homogeneous series of platinum-resistant patients who underwent a maximum of two lines of previous chemotherapy treatment (ClinicalTrials.gov identifier: NCT02735928). Our study shows that PIPAC with intraperitoneal cisplatin and doxorubicin is active and well tolerated in women with platinum-resistant ovarian cancer, with 82% of patients achieving a CBR.

Regarding the recent literature data on PIPAC,^47–51^ the strength of this manuscript lies in the fact that it is the first phase II trial on women with the same advanced disease (i.e., ovarian cancer), the biological characteristics (i.e., platinum-resistant), and the same number of previous lines of chemotherapies administered. Moreover, while the trial was ongoing, although different authors^49,50^ performed dose-escalation studies that highlighted that PIPAC could be performed at the highest dosage, we decided to maintain the exact dosage according to Tempfer at al.^34^ to guarantee more homogeneous results. Moreover, the reason for the limited cycle of PIPAC in 55% of patients was due to disease progression documented with a radiological instrumental examination or for worsening of clinical conditions. Indeed, we should consider the unfavorable setting of women in this trial (platinum-resistant after only one to two lines of chemotherapy) with respect to other experiences.^22,47,48^

Our results agree with the preliminary, and still unpublished, results of the Indian phase II study that was presented in poster format at ASCO in 2022, in which PIPAC was shown to be safe and feasible for patients with unresectable platinum-resistant ovarian cancer.^47^ Somashekhar et al.^48^ have already published data regarding the application of PIPAC in patients affected by gynecological and gastrointestinal tumors with end-stage peritoneal metastasis. They showed a promising response, a good tolerance profile, and QoL after three cycles of PIPAC in comparison with six cycles of systemic chemotherapy in this inhomogeneous cohort of patients, using the EORTC QLQ-C30 (version 3.0) questionnaire.

Of note, overall QoL scores for global physical health scores do not decrease during therapy. An objective minor tumor response and no tumor response with SD was documented in 29.6% and 70.4% of patients, respectively, after two PIPAC-directed treatments. The median overall survival after the first diagnosis was 27.0 months, while time to progression after the first PIPAC procedure was 12 months. These results agree well with the recently presented experiences from German centers and are consistent with previous experience with PIPAC in patients with platinum-resistant recurrence.^52^

Until now, anecdotal evidence^12^ and retrospective case series^15^ demonstrated objective tumor response and histological tumor regression in ovarian cancer, with acceptable local and systemic toxicity. This is the first prospective report that seems to confirm these results in a homogeneous clinical subset of patients.

Our data confirm that PIPAC is safe and well tolerated in women with platinum-resistant ovarian cancer, even if applied without concomitant cytoreductive surgery. Moreover, bearing in mind that the administration of some drugs (i.e., bevacizumab) is associated with a similar bowel perforation rate without surgery, the PIPAC would not replace them but would integrate itself with the timing of other drugs administered in this subset of patients.

Of note, most women in our trial were pretreated with a median number of one prior chemotherapy regimens, indicating that PIPAC may be able to overcome platinum resistance at least in a fraction of women. This may be related to the high local chemotherapy concentrations achieved by the intra-abdominal application, and/or by the better uptake of the drug induced by the peritoneal vasodilation caused by the hypercapnic intraperitoneal environment of the pneumoperitoneum.^17^ Another factor may be the chemical peritonitis induced by intra-abdominal application of the cytotoxic drugs themselves.^53^ A potential beneficial aspect of PIPAC compared with systemic cytotoxic chemotherapy may be its adverse effect profile. In order to objectify the impact of PIPAC on the QoL of study participants, we have systematically measured QoL scores using the EORTC QLQ-30 and QLQ-OV28 questionnaires. It is of note that gastrointestinal scores such as those for nausea/vomiting, appetite loss, and constipation slightly decreased during therapy, which could indicate that PIPAC stabilizes peritoneal carcinosis and leads to an improvement of bowel function. In addition, physical, cognitive, and emotional scores improved and fatigue scores decreased during therapy, supporting the notion that the women experienced a clinical benefit from PIPAC. Notorious systemic adverse effects of chemotherapy, such as alopecia, peripheral neurotoxicity, nausea, and myelosuppression, did not occur with PIPAC.

This phase II trial has some limitations. First, patients were highly selected; for example, women with liver and lung metastases were excluded, and it is possible that patients with the best prognosis were thus selected. However, our main goal was to report the feasibility of procedures in an homogeneous setting of women. Moreover, although, as a monocentric trial, a weakness could lie in the lack of variation of the surgical experience in different institutes, at the same time it offers the surgical background of a single center where surgical care and laparoscopic evaluation are standardized, overcoming any difference such as preoperative patient selection, surgical strategies, and postoperative management.

Second, the small number of patients in this phase II trial may obscure rare adverse events and toxicities. Furthermore, we have no long-term follow-up data and cannot rule out late adverse events such as bowel sclerosis. Third, although no clinical decision was performed based on Fagotti’s score, since it has been developed as a staging tool for peritoneal carcinomatosis and not as a measure of therapy response, we did not observe any regressions in terms of peritoneal dissemination, but this evaluation could be incorrect. Lastly, pathological response assessment may be inaccurate because it is difficult to differentiate between scars and vital tumor tissue, and, even if we use a landmark for peritoneal biopsy, the heterogeneity of the peritoneal carcinosis could not mirror the response to chemotherapy.

In summary, PIPAC seems to play an active role in platinum-resistant ovarian cancer recurrence, is easy to perform, and is well tolerated if strict selection of patients was performed. PIPAC is not associated with a decrease in QoL. Whether or not PIPAC is a clinically meaningful therapeutic option in the setting of palliative ovarian cancer treatment remains to be seen.

Further comparative clinical trials testing the efficacy of PIPAC versus, or in addition to, systemic chemotherapy are warranted, thus making it possible to treat women with extra-abdominal disease. Indeed, this paper is only the first mandatory step to pave the subsequent scientific trials, trying to extend the applications of PIPAC with or without concomitant chemotherapy in different subsets of women with ovarian cancer, choosing the right dosage and timing.

Thus, we consider that our study may be hypothesis-generating since the potential role of other chemotherapy compounds such as taxanes, topotecan, gemcitabine, and/or nanoparticles should be investigated in PIPAC protocols in this or different subsets of patients. Indeed, since the platinum-resistant nature of the relapse, these agents may be more effective and PIPAC should be intended as a new different drug delivery system.

Finally, combining cytotoxic agents with bevacizumab and/or its role in the poly(ADP-ribose) polymerase (PARP) inhibitor era with or without BRCA mutation may be an attractive option for future PIPAC trial designs.

### Supplementary Information

Below is the link to the electronic supplementary material.

Supplementary file1 (DOCX 18 KB)
