# Supplementary material for: Pressurized IntraPeritoneal Aerosol Chemotherapy (PIPAC) Applied to Platinum-Resistant Recurrence of Ovarian Tumor: A Single-Institution Experience (ID: PARROT Trial)
Source: Ann Surg Oncol. 2023 Dec 15;31(2):1207–16. doi: 10.1245/s10434-023-14648-0 (PMC10761392; doi:10.1245/s10434-023-14648-0)
Supplement: Supplementary file 1 — Supplementary file1 (DOCX 18 KB) [file 10434_2023_14648_MOESM1_ESM.docx]

**Suppl. table 1. Mean scores and standard deviations of the EORTC QLQ C-30 scales by treatment and assessment time.**

| **Scale/item** | **Baseline/ PIPAC #1*** | **PIPAC #2*** | **PIPAC #3*** | **p value^§^** |
| --- | --- | --- | --- | --- |
| *Global health status* | 63.89 (±3.25) | 41.67 (±3.15) | 62.00 (±3.03) | 0.347 |
| *Physical functioning* | 77.59 (±3.26) | 88.57 (±4.66) | 90.64 (±5.77) | 0.231 |
| *Role functioning* | 58.37 (±4.84) | 56.65 (±3.54) | 68.75 (±3.87) | 0.291 |
| *Emotional functioning* | 66.11 (±4.33) | 56.25 (±4.41) | 58.33 (±3.55) | 0.336 |
| *Cognitive functioning* | 89.44 (±2.58) | 95.83 (±4.16) | 95.45 (±4.54) | 0.351 |
| *Social functioning* | 75.92 (±3.89) | 71.84 (±4.84) | 70.04 (±4.30) | 0.380 |
| *Fatigue* | 34.81 (±4.21) | 31.25 (±3.37) | 38.90 (±3.45) | 0.477 |
| *Nausea and vomiting* | 12.78 (±3.54) | 23.06 (±4.01) | 11.87 (±4.84) | 0.410 |
| *Pain* | 26.67 (±3.79) | 40.73 (±4.89) | 43.75 (±6.07) | 0.423 |
| *Dyspnea* | 33.71 (±3.65) | 25.27 (±3.99) | 16.22 (±3.51) | 0.363 |
| *Insomnia* | 23.33 (±3.25) | 22.40 (±4.37) | 25.75 (±3.85) | 0.644 |
| *Appetite loss* | 21.11 (±3.74) | 12.69 (±4.35) | 10.47 (±2.88) | 0.168 |
| *Constipation* | 43.16 (±5.09) | 58.65 (±5.02) | 62.63 (±4.26) | 0.185 |
| *Diarrhea* | 4.44 (±2.10) | 6.06 (±4.02) | 6.29 (±2.27) | 0.233 |
| *Financial difficulties* | 22.22 (±4.32) | 37.18 (±5.07) | 37.71 (±4.66) | 0.464 |

* All results are expressed as mean±standard error (SE)

**^§^** p value is referred to Anova one way variance test

**Suppl. table 2. Mean scores and standard deviations of the EORTC QLQ OV-28 scales by treatment and assessment time.**

| **Scale/item** | **Baseline/ PIPAC #1*** | **PIPAC #2*** | **PIPAC #3*** | **p value^§^** |
| --- | --- | --- | --- | --- |
| *Abdominal/GI symptoms* | 29.81 (± 2.91) | 33.98 (± 2.85) | 35.96 (±2.95) | 0.735 |
| *Hair loss* | 13.02 (± 2.24) | 11.18 (± 3.68) | 11.65 (±3.49) | 0.813 |
| *Peripheral neuropathy* | 11.57 (± 2.91) | 18.20 (± 2.44) | 11.39 (±3.45) | 0.390 |
| *Other chemotherapy side effects* | 14.66 (± 2.77) | 12.24 (± 2.43) | 11.45 (±2.85) | 0.683 |
| *Hormonal/menopausal symptoms* | 13.35 (± 2.99) | 16.96 (± 2.61) | 14.12 (±3.44) | 0.733 |
| *Body image* | 34.48 (± 5.41) | 38.89 (± 4.29) | 25.26 (±4.57) | 0.407 |
| *Attitude to disease/treatment* | 63.70 (± 3.64) | 67.72 (± 3.26) | 61.91 (±3.49) | 0.874 |
| *Sexuality* | 4.58 (± 2.20) | 3.86 (± 1.79) | 3.69 (±1.63) | 0.841 |

* All results are expressed as mean±standard error (SE)

**^§^** p value is referred to Anova one way variance test
